# Whole genome sequencing and phylogenetic analysis of SARS-CoV-2 strains isolated during the COVID-19 pandemic in Nigeria

**DOI:** 10.1016/j.ijregi.2024.01.005

**Published:** 2024-01-17

**Authors:** Adedolapo B. Olorunfemi, Salma A.R. Suliman, Tung T. Tran, Babatunde Ayorinde, Muinah A. Fowora, Bamidele A. Iwalokun, Olugbenga A. Olowe, Oluyinka O. Opaleye, Mohamed Osman, Babatunde L. Salako, Richard Adegbola, Bolaji N. Thomas, Srinivas Reddy Pallerla, Thirumalaisamy P. Velavan, Olusola Ojurongbe

**Affiliations:** 1 Center for Emerging and Re-emerging Infectious Diseases, Ladoke Akintola University of Technology, Ogbomoso, Nigeria; 2 Department of Medical Microbiology and Parasitology, Ladoke Akintola University of Technology, Ogbomoso, Nigeria; 3 Institute of Endemic Diseases, University of Khartoum, Sudan; 4 Vietnamese-German Center for Medical Research (VG-CARE), Hanoi, Vietnam; 5 Molecular Biology & Biotechnology Department, Nigerian Institute of Medical Research (NIMR), Yaba, Lagos, Nigeria; 6 Central Research Laboratory, Nigerian Institute of Medical Research (NIMR), Yaba, Lagos, Nigeria; 7 York Biomedical Research Institute, Department of Biology, University of York, Wentworth Way, York YO10 5DD, UK; 8 Department of Biomedical Sciences, College of Health Sciences and Technology, Rochester Institute of Technology, Rochester, NY 14623, USA; 9 Institute for Medical Virology, University Hospital Frankfurt, Goethe University Frankfurt, Frankfurt, Germany; 10 Institute of Tropical Medicine, Universitätsklinikum Tübingen, Universität Tübingen, Germany

**Keywords:** SARS-CoV-2, COVID-19, Whole genome sequencing, MinION, Pandemic, Nigeria

## Abstract

**Objectives:**

The emergence and spread of SARS-CoV-2 have stimulated ongoing research into the virus transmission dynamics, circulating variants, and potential mutations. This study was conducted to understand the genomic dynamics of the epidemic in Nigeria.

**Design:**

Whole genome sequencing was conducted on SARS-CoV-2 samples collected during the first and second outbreaks using the Oxford Nanopore MinION sequencing platform. Phylogenetic analysis was conducted, and genomes were grouped into different pangolin lineages.

**Results:**

The study revealed four circulating SARS-CoV-2 variants. The Alpha (B.1.1.7) variant was the most prevalent (32.7%), followed by Beta (B.1 B.1.1, L.3, and B.1.1.318) (30.8%), Eta (B.1.525) (28.9%), and Delta (B.1.617, AY.1, AY.109, and AY.36) (7.7%). Phylogenetic analysis revealed three clusters with four Nextstrain clades (20I, 20B, 21D, and 21J). The Alpha lineages (B.1.1.7) clustered with references from Italy. The Beta lineages (Clade 20B) (B.11, B.11318, and L3) and sub-lineage B.11 were distinct. Sub-lineage B.11318 is clustered with references from the USA, whereas sub-lineage L3 is clustered with references from Russia, the Philippines, Australia, and Japan. The 21D and 21J, belonging to two Pango lineages, Eta (B.1525) and Delta (B.1.617 and AY.109), showed high genetic similarity.

**Conclusion:**

The phylogenetic relatedness of the lineages suggests multiple virus introduction, which could be a source of more virulent, locally adapted variants.

## Highlights

• The Alpha, Beta, Eta, and Delta SARS-CoV-2 variants were in circulation in Nigeria.
• A greater incidence of Eta lineage of SARS-CoV-2 was observed in Nigeria.
• Low mortality may have impacted the COVID-19 vaccine receptibility in Nigeria.
• Phylogenetic relatedness of SARS-CoV-2 lineages indicates multiple introductions.

## Introduction

COVID-19, caused by SARS-CoV-2 infection, was first identified in Wuhan, China in December 2019 and declared a pandemic by the World Health Organization on March 11, 2020 [1,2]. Within a short period, the infection spread from China to about 215 countries and regions, including Africa. There have been 759,408,703 confirmed cases of COVID-19 as of March 7, 2023, with 6,866,434 fatalities worldwide [3].

The first confirmed case of COVID-19 in Nigeria was announced on February 27, 2020, when an infected traveler from Italy arrived in Lagos by commercial aircraft [4,5]. The official data, according to the World Health Organization, as of 7 January 19, 2024, showed 267,173 confirmed cases and 3155 fatalities in Nigeria [3], indicating the continuous spread of the virus. Introducing SARS-CoV-2 to a new region often causes sporadic outbreaks, necessitating urgent testing, tracing, and epidemiological investigations [6]. To better understand the transmission dynamics of the virus, its genetic alterations, and its evolution and to make policy decisions toward combating the pandemic, rapid, cost-effective, and real-time genome sequencing of the virus combined with relevant epidemiological data are essential resources [7]. Understanding the transmission patterns of epidemics and informing choices about outbreak management are some of the many benefits of whole genome sequencing (WGS) [8,9].

During the 2014-2016 West African Ebola epidemic, real-time WGS aided public health decision-making as part of a strategy known as precise public health pathogen genomes [10]. After the outbreak of COVID-19, WGS of the SARS-CoV-2 virus has played an important role, including evolutionary analysis of the virus, monitoring of circulating genetic lineages, and finding signs of adaptation to hosts, which have important implications for treatment and vaccine development [11,12]. The timely use of SARS-CoV-2 WGS directly affects local infection prevention and control actions based on high-resolution genomic data. Understanding how viruses spread is crucial for clinical and public health decisions. SARS-CoV-2 tends to evolve rapidly, so monitoring the emergence of variants of concern is crucial to assessing the efficiency of antiviral medications, diagnostic tests, and vaccines [13]. Using the Oxford Nanopore MinION, this study, therefore, performed a WGS of SARS-CoV-2 viruses collected during the first and second waves of the COVID-19 pandemic in Nigeria. Furthermore, the phylogenetic analysis compared the Nigerian SARS-CoV-2 strain to other publicly available genomes worldwide.

## Materials and methods

### Study population and sample collection

The study samples were collected between August 2020 and October 2021. The samples were collected during surveillance, including routine screening for influenza-like illnesses and targeted surveillance of COVID-19 cluster outbreaks in Lagos and Oyo States. The nasopharyngeal specimen was collected by carefully inserting a sterile swab into the nostril parallel to the palate for a few seconds to absorb secretion. The swab was then placed in a viral transport medium tube and stored at −20°C until use. Samples were screened and sequenced at the Humboldt Research Hub-Centre for Emerging and Re-emerging Infectious Diseases, Ladoke Akintola University of Technology, Ogbomoso, Nigeria.

### RNA extraction and reverse transcription-polymerase chain reaction confirmation

Viral RNA was extracted from all samples using the QIAamp Viral RNA Extraction Kit (Qiagen Mini kit) according to the manufacturer's instructions. A total of 60 μL of purified RNA was eluted and kept at −80°C until further use. Quantitative real-time reverse transcription-polymerase chain reaction was used to screen the samples collected to determine positive samples and their cycle threshold (Ct) using the GENESIG Real-Time PCR COVID-19CE IVD Kit (Primer design, Chandler's Ford, UK) according to the manufacturer's specification. Reactions were run at a total volume of 20 µL on QuantStudio™ 5 RT-PCR System (Applied Biosystems, Waltham, MA, USA). The samples for WGS were selected based on SARS-CoV-2 RNA concentration measured by Ct values ≤30.

### Complimentary DNA and amplicon generation

Reverse transcription of RNA samples with a Ct value ≤30 was performed using the LunaScript RT SuperMix kit (New England Biolabs, Ipswich, MA, USA). Briefly, 8 μL RNA was mixed with 2 μL LunaScript RT SuperMix (5 ×), placed in a thermocycler (Eppendorf, Hamburg, Germany), and incubated for 2 minutes at 25°C, followed by 10 minutes at 55°C and 1 minute at 95°C and cooling to 4°C. Two independent PCR reactions were carried out for each sample, with 58 primers in pools 1 and 2 containing 29 primers each, yielded as odd- and even-numbered tiled amplicons for the 1200 bp set (Supplementary Tables 1 and 2), as previously described [14].

### Library preparation and sequencing using Oxford Nanopore Technology

The two PCR products were pooled, and a library was prepared using an Oxford Nanopore Technologies Rapid Barcoding Kit (SQK-RBK110.96). Before the library preparation, the PCR products were purified using a 1:1 ratio of AMPure XP Beads (Beckman Coulter Diagnostics, Brea, CA, USA). Quantification (Qubit DNA BR, Thermo Scientific), normalization, barcoding of each sample, pooling of the barcoded samples, and purification (using AMPure XP Beads) of the pooled samples were performed at 1:1. The libraries were then loaded on the Oxford Nanopore MinION SpotON R9.4 Flow Cell. After sequencing, only samples that generated complete genome coverage were used for further analyses.

### Base-calling, assembly, and phylogenetic analysis

The Fast5 files generated during sequencing were basecalled to FastQ files using Guppy v.3.4.5 (Oxford Nanopore Technologies) on the MinION IT device. The COVID-19 bioinformatics Medaka pipeline developed by the ARTIC network (https://artic.network/ncov-2019/ncov2019-bioinformatics-sop.html) was used to generate consensus sequences and call variant nucleotides (pangolin lineages) relative to the reference sequence from Wuhan-Hu-1 (accession NC_045512). Regions with >10-fold coverage were used to determine the genome coverage percentages.

A multiple sequence alignment of the sequenced samples was done using MEGA 11, and the multiple sequence alignment was carefully examined to filter out any poor-quality sequences. SARS-CoV-2 genome sequences from various nations, including NC 045512.2-Wuhan-Hu-,1, were retrieved from the National Center for Biotechnology Information and compared with our sequences. The comparisons generated a maximum likelihood and evolutionary tree using Fast Tree v2.1.11 (http://www.microbesonline.org/fasttree/).

## Results

### Characterization of SARS-CoV-2 isolates from Nigeria

A total of 82 samples were positive for SARS-CoV-2 when screened using quantitative real-time reverse transcription-polymerase chain reaction, of which 52 samples with Ct value ≤30 were selected for sequencing using the MinION device. Of the 52 samples chosen for sequencing, 34 (65.4%) represented male participants, and 18 (34.6%) represented female participants. The average age of the patients was 38 years (range 15-80 years). Most of the positive samples collected were from hospitalized patients (59.6%), whereas others were from outpatients (40.4%) (Table 1). The percentage genome coverage ranges from 82.0-99.6%, with an average genome coverage of 85.6%.

**Table 1:** Demographic characteristics of tested patients.

Parameters	Samples (n = 52)	Overall (%)
**Age groups (years)**		
15-29	10	19.2
30-49	24	46.2
50-80	17	34.6
**Gender**		
Male	34	65.4
Female	18	34.6
**Hospitalization**		
On-Admission	31	59.6
Not Admitted	21	40.4

**Figure 1:**
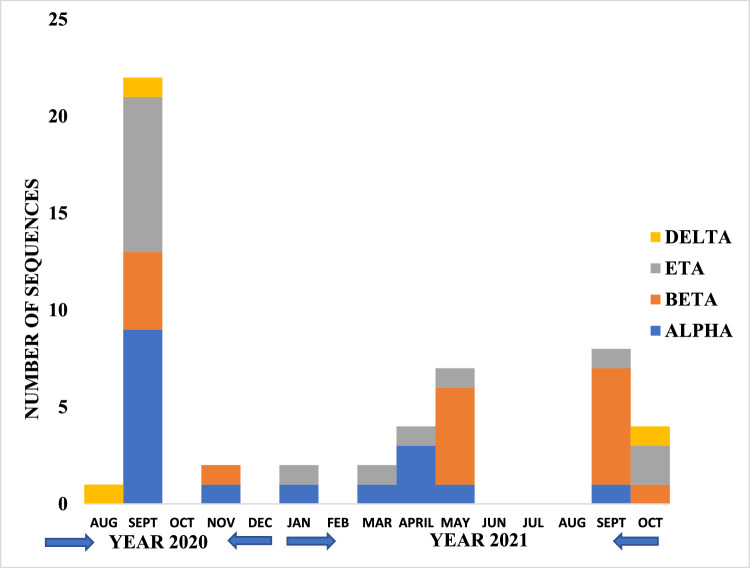
Distribution of SARS-CoV-2 lineages in Nigeria; August 2020-October 2021.

The sequenced SARS-CoV-2 genomes and their respective viral lineages with respect to the period of the collection are summarized in Figure 1. The sequences were clustered in four Pango lineages (Alpha, Beta, Eta, and Delta). Alpha (B.1.1.7) was the most predominant, with 32.7%, followed by Beta (B.1 B.1.1, L.3, and B.1.1.318) with 30.8%, and Eta (B.1.525) with 28.9%. The Delta variant (B.1.617, AY.1, AY.109, and AY.36) had the lowest prevalence of 7.7%. Most predominant Pango lineages were observed among hospitalized male patients (19.2%). The observed lineages from the first and the second waves were similar. Out of the 52 samples, 23 (44.2%) sequences met the quality criteria (% genome length of more than 90, the number of ambiguous reads <3000) and were chosen for phylogenetic analysis (Supplementary Table 3). The SARS-CoV-2 genome sequences were submitted to the Global Initiative on Sharing All Influenza Data genome bank (https://gisaid.org/) with the proper metadata.

### Phylogenetic analysis of SARS-CoV-2 from Nigeria

**Figure 2:**
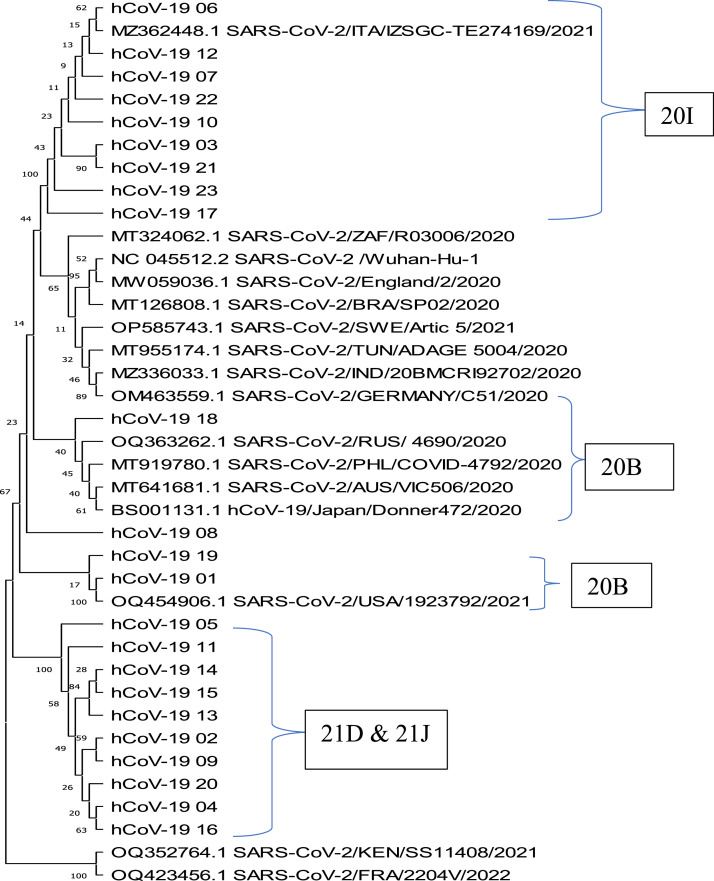
Phylogenetic tree showing the genetic relatedness of SARS-CoV-2 lineages in Nigeria compared with reference sequences, using the maximum likelihood statistical method with 500 bootstrap replications.

The analysis revealed the presence of four distinct clusters of the selected SARS-CoV-2 samples, with four Nextstrain clades (20I, 20B, 21D, and 21J) (Figure 2). The first clade, 20I, belonging to the Alpha lineages (B.1.1.7), was found in nine isolates and clustered with references from Italy (MZ362448.1). The second clade, 20B, comprises four isolates and belongs to the Beta lineages (B.11, B.11318, and L3). Sub-lineage L3 clustered with references from Russia (OQ363262.1), Philippines (MT919780.1), Australia (MT641681.1), and Japan (BS001131.1). Sub-lineage B.11 was found to be isolated from the rest of the samples and downloaded references, whereas sub-lineage B.11318 clustered with references from the USA (OQ454906.1). The last two clades, 21D and 21J, comprise 10 isolates and belong to two Pango lineages, Eta (B.1525) and Delta (B.1.617 and AY.109), respectively. They showed high of genetic similarity, clustering alone, and isolated from the rest of the samples and downloaded references. However, some downloaded reference sequences did not cluster with any of the sequences analyzed in this study.

## Discussion

Although confirmed COVID-19 cases and fatalities are decreasing worldwide, more research into the virus is needed to better understand its possible re-emergence and make plans to control future pandemics. This study contributes to temporal and geographical SARS-CoV-2 surveillance data by providing basic information on the virus’ transmission in Nigeria between August 2020 and October 2021, representing the first and second waves. Four major Pango lineages, designated as Alpha, Beta, Eta, and Delta, were found to be spreading during this period. The spread of the virus within these states is most likely owing to the frequent movement of people in and out of the states. The most predominant lineage (Alpha) observed in our study was first reported on December 21, 2020 in the UK, representing a significant percentage of cases reported in most of Nigeria by early January 2021 [15]. Because Lagos is the gateway into the country, it is unsurprising that this variant seems to predominate in other parts of the country, including Oyo State, which shares a boundary with Lagos State [15], [16]. Although this lineage does not affect the efficacy of neutralizing antibodies produced by vaccination, it has been shown to be more infectious *in vitro*, implying increased transmissibility [17] and the ability to interfere with diagnostic test probing, a distinctive trait of the Alpha variant [18,19]. Because B.1.1.7 is thought to have a greater infectivity rate and antibodies no longer successfully neutralize E484K variants, this lineage could likely be partly responsible for a significant increase in daily occurrence observed in Nigeria in late 2020.

Similar to the Alpha lineage, the Eta lineage (B.1.525) was detected in Nigeria in the fall of 2020, shortly after lifting travel restrictions [20]. As a result of their imports and subsequent spread, these two variants predominated in the country by early 2021 [16]. As of February 14, 2021, approximately 25% of all alleles in the Global Initiative on Sharing All Influenza Data originated from Nigeria, making it the nation with the most significant frequency of the B.1.525 lineage [21]. Some studies have shown that the spike E484K mutation in B.1.525 may affect immune recognition and vaccination effectiveness [22,23]. Most of the Delta isolates (7.7%) in this study were from the uncommon lineages (B.1.617, AY.1, AY.109, and AY.36), which only made up 0.5% of all Delta sequences worldwide. Delta variants become the most prevalent in the country by June 2021; however, they still account for a fraction of all cases. The B.1.617 lineage emerged in India in October 2020 and subsequently spread to the UK and the USA. Three notable substitutions in the spike protein (L452R, E484Q, and P681R) present in this lineage may lower neutralization from antibodies, implying that the lineage can escape immune response or become resistant to control measures, including vaccination [24]. Unfortunately, the low mortality rate and the myth surrounding COVID-19 [25] may have contributed significantly to Nigeria's low vaccine coverage, making it impossible to accurately depict the effect of the spike mutations observed in the different variants on vaccine effectiveness in Nigeria.

The phylogenetic analyses demonstrate the dominance of multiple distinct SARS-CoV-2 lineages in Nigeria, suggesting a separate introduction of the virus into the country. The Alpha (B.1.1.7) lineage isolates in this study were found to be monophyletic and genetically linked to the reference sequence from Italy, implying a single introduction into the country. The first confirmed case of COVID-19 in Nigeria was announced when an infected traveler from Italy arrived by commercial aircraft in Lagos on the February 25, 2020. This may confirm the successful entry of the Alpha lineage from Italy into the country. The close relatedness of the Beta lineages with four countries (Russia, Philippines, Australia, Japan, and the USA) indicates multiple successful introductions of this lineage into the country. The relaxation of travel restrictions in the autumn of 2020 might have paved the way for these introductions. Sub-lineage B.11 was discovered to be distinct from the rest of the samples and retrieved references suggested this may have been a local adaptation. Similarly, the Eta (B.1525) and Delta (B.1.617 and AY.109) lineages were also found to be isolated from the rest of the samples and downloaded references. These isolated populations may be a result of genetic changes that are not shared with the rest of the samples, leading to the formation of distinct subpopulations with their own evolutionary trajectory. It may be necessary to monitor this seemingly locally adapted variant to understand its infectivity, transmissibility, and disease severity pattern. The successful entry and spread of the Eta lineage at a very low rate and their possible fitness advantages may be attributed to their observed prevalence in Nigeria [15].

## Conclusion

This study revealed the circulation of four SARS-CoV-2 variants (Alpha, Beta, Eta, and Delta) in Nigeria during the first and second waves of COVID-19. In comparison to other nations, Nigeria had a greater incidence of Eta lineage observed in this study. SARS-CoV-2 samples must be sequenced and monitored regularly across Nigeria to detect and respond to emerging variants. As vaccination efforts continue, monitoring these lineages and understanding how the spike mutation in B.1.525 (E484K) and B.1.617 (L452R, E484Q, and P681R) may affect immune recognition and vaccination effectiveness is vital. Considering that this epidemic is unlikely to be the last, public health systems must improve their ability to respond and establish and sustain robust scientific research initiatives.

## Declarations of competing interest

The authors have no competing interests to declare.

## Appendix Supplementary materials

Image, application 1
