# Supplementary material for: Whole genome sequencing and phylogenetic analysis of SARS-CoV-2 strains isolated during the COVID-19 pandemic in Nigeria
Source: IJID Reg. 2024 Jan 17;10:174–8. doi: 10.1016/j.ijregi.2024.01.005 (PMC10845906; doi:10.1016/j.ijregi.2024.01.005)
Supplement: Supplementary file 1 [file mmc1.docx]

**Supplementary Tables**

**Table 1:** **List of primers used for multiplex PCR and their genomic locations (PCR- Pool (1) Primers)**

| **Primer Name** | **Sequence** | **Start** |
| --- | --- | --- |
| SARSCoV_1200_1_LEFT | ACCAACCAACTTTCGATCTCTTGT | 30 |
| SARSCoV_1200_1_RIGHT | GGTTGCATTCATTTGGTGACGC | 1205 |
| SARSCoV_1200_3_LEFT | GGCTTGAAGAGAAGTTTAAGGAAGGT | 2153 |
| SARSCoV_1200_3_RIGHT | GATTGTCCTCACTGCCGTCTTG | 3257 |
| SARSCoV_1200_5_LEFT | ACCTACTAAAAAGGCTGGTGGC | 4167 |
| SARSCoV_1200_5_RIGHT | AGCATCTTGTAGAGCAGGTGGA | 5359 |
| SARSCoV_1200_7_LEFT | ACCTGGTGTATACGTTGTCTTTGG | 6283 |
| SARSCoV_1200_7_RIGHT | GCTGAAATCGGGGCCATTTGTA | 7401 |
| SARSCoV_1200_9_LEFT | AGAAGTTACTGGCGATAGTTGTAATAACT | 8253 |
| SARSCoV_1200_9_RIGHT | TGCTGATATGTCCAAAGCACCA | 9400 |
| SARSCoV_1200_11_LEFT | AGACACCTAAGTATAAGTTTGTTCGCA | 10343 |
| SARSCoV_1200_11_RIGHT | GCCCACATGGAAATGGCTTGAT | 11469 |
| SARSCoV_1200_13_LEFT | ACCTCTTACAACAGCAGCCAAAC | 12450 |
| SARSCoV_1200_13_RIGHT | CGTCCTTTTCTTGGAAGCGACA | 13621 |
| SARSCoV_1200_15_LEFT | TTTTAAGGAATTACTTGTGTATGCTGCT | 14540 |
| SARSCoV_1200_15_RIGHT | ACACACAACAGCATCGTCAGAG | 15735 |
| SARSCoV_1200_17_LEFT | TCAAGCTTTTTGCAGCAGAAACG | 16624 |
| SARSCoV_1200_17_RIGHT | CCAAGCAGGGTTACGTGTAAGG | 17754 |
| SARSCoV_1200_19_LEFT | GGCACATGGCTTTGAGTTGACA | 18596 |
| SARSCoV_1200_19_RIGHT | CCTGTTGTCCATCAAAGTGTCCC | 19678 |
| SARSCoV_1200_21_LEFT | TCTGTAGTTTCTAAGGTTGTCAAAGTGA | 20553 |
| SARSCoV_1200_21_RIGHT | GCAGGGGGTAATTGAGTTCTGG | 21642 |
| SARSCoV_1200_23_LEFT | ACTTTAGAGTCCAACCAACAGAATCT | 22511 |
| SARSCoV_1200_23_RIGHT | TGACTAGCTACACTACGTGCCC | 23631 |
| SARSCoV_1200_25_LEFT | TGCTGCTACTAAAATGTCAGAGTGT | 24633 |
| SARSCoV_1200_25_RIGHT | CATTTCCAGCAAAGCCAAAGCC | 25790 |
| SARSCoV_1200_27_LEFT | TGGATCACCGGTGGAATTGCTA | 26744 |
| SARSCoV_1200_27_RIGHT | TGTTCGTTTAGGCGTGACAAGT | 27894 |
| SARSCoV_1200_29_LEFT | TGAGGGAGCCTTGAATACACCA | 28677 |
| SARSCoV_1200_29_RIGHT | TAGGCAGCTCTCCCTAGCATTG | 29790 |

**Table 2: PCR- Pool (2) Primers**

| Primer Name | Sequence | Start |
| --- | --- | --- |
| SARSCoV_1200_2_LEFT | CCATAATCAAGACTATTCAACCAAGGGT | 1100 |
| SARSCoV_1200_2_RIGHT | ACAGGTGACAATTTGTCCACCG | 2266 |
| SARSCoV_1200_4_LEFT | GGAATTTGGTGCCACTTCTGCT | 3144 |
| SARSCoV_1200_4_RIGHT | CCTGACCCGGGTAAGTGGTTAT | 4262 |
| SARSCoV_1200_6_LEFT | ACTTCTATTAAATGGGCAGATAACAACTG | 5257 |
| SARSCoV_1200_6_RIGHT | GATTATCCATTCCCTGCGCGTC | 6380 |
| SARSCoV_1200_8_LEFT | CAATCATGCAATTGTTTTTCAGCTATTTTG | 7298 |
| SARSCoV_1200_8_RIGHT | TGACTTTTTGCTACCTGCGCAT | 8385 |
| SARSCoV_1200_10_LEFT | TTTACCAGGAGTTTTCTGTGGTGT | 9303 |
| SARSCoV_1200_10_RIGHT | TGGGCCTCATAGCACATTGGTA | 10451 |
| SARSCoV_1200_12_LEFT | ATGGTGCTAGGAGAGTGTGGAC | 11372 |
| SARSCoV_1200_12_RIGHT | GGATTTCCCACAATGCTGATGC | 12560 |
| SARSCoV_1200_14_LEFT | ACAGGCACTAGTACTGATGTCGT | 13509 |
| SARSCoV_1200_14_RIGHT | GTGCAGCTACTGAAAAGCACGT | 14641 |
| SARSCoV_1200_16_LEFT | ACAACACAGACTTTATGAGTGTCTCT | 15608 |
| SARSCoV_1200_16_RIGHT | CTCTGTCAGACAGCACTTCACG | 16720 |
| SARSCoV_1200_18_LEFT | GCACATAAAGACAAATCAGCTCAATGC | 17622 |
| SARSCoV_1200_18_RIGHT | TGTCTGAAGCAGTGGAAAAGCA | 18706 |
| SARSCoV_1200_20_LEFT | ACAATTTGATACTTATAACCTCTGGAACAC | 19574 |
| SARSCoV_1200_20_RIGHT | GATTAGGCATAGCAACACCCGG | 20698 |
| SARSCoV_1200_22_LEFT | GTGATGTTCTTGTTAACAACTAAACGAACA | 21532 |
| SARSCoV_1200_22_RIGHT | AACAGATGCAAATCTGGTGGCG | 22612 |
| SARSCoV_1200_24_LEFT | GCTGAACATGTCAACAACTCATATGA | 23518 |
| SARSCoV_1200_24_RIGHT | ATGAGGTGCTGACTGAGGGAAG | 24736 |
| SARSCoV_1200_26_LEFT | GCCTTGAAGCCCCTTTTCTCTA | 25690 |
| SARSCoV_1200_26_RIGHT | AATGACCACATGGAACGCGTAC | 26857 |
| SARSCoV_1200_28_LEFT | TTTGTGCTTTTTAGCCTTTCTGCT | 27784 |
| SARSCoV_1200_28_RIGHT | GTTTGGCCTTGTTGTTGTTGGC | 29007 |

*Positions relative to MN908947/SARS-CoV-2/Wuhan-Hu-1

**Table 3: Samples selected for phylogenetic analysis and their Pango Lineages based on the genome length**

| **Sample ID** | **Genome Coverage (%)** | **Pango Lineages** |
| --- | --- | --- |
| hcov-19-01 | 99.4 | B.1.617 |
| hcov-19-02 | 98.2 | B.1.525 |
| hcov-19-03 | 98.6 | B.1.1.7 |
| hcov-19-04 | 98.4 | B.1.525 |
| hcov-19-05 | 96.7 | B.1.617 |
| hcov-19-06 | 97.8 | B.1.1.7 |
| hcov-19-07 | 99.8 | B.1.1.7 |
| hcov-19-08 | 98.6 | B.1.1 |
| hcov-19-09 | 99.7 | B.1.525 |
| hcov-19-10 | 98.3 | B.1.1.7 |
| hcov-19-11 | 97.3 | B.1.617 |
| hcov-19-12 | 96.8 | B.1.1.7 |
| hcov-19-13 | 98.5 | B.1.617 |
| hcov-19-14 | 99.6 | B.1.617 |
| hcov-19-15 | 98.4 | B.1.525 |
| hcov-19-16 | 97.5 | B.1.525 |
| hcov-19-17 | 100 | B.1.1.7 |
| hcov-19-18 | 97.6 | L3 |
| hcov-19-19 | 99.9 | B.1.1.318 |
| hcov-19-20 | 98.8 | AY.109 |
| hcov-19-21 | 99.5 | B.1.1.7 |
| hcov-19-22 | 96.8 | B.1.1.7 |
| hcov-19-23 | 99.6 | B.1.1.7 |
